# An In Vitro Study of the Influence of *Curcuma longa* Extracts on the Microbiota Modulation Process, In Patients with Hypertension

**DOI:** 10.3390/pharmaceutics11040191

**Published:** 2019-04-18

**Authors:** Emanuel Vamanu, Florentina Gatea, Ionela Sârbu, Diana Pelinescu

**Affiliations:** 1 Faculty of Biotechnology, University of Agronomic Science and Veterinary Medicine, 59 Marasti blvd, 1 district, 011464 Bucharest, Romania; 2 Centre of Bioanalysis, National Institute for Biological Sciences, 296 Spl. Independentei, 060031 Bucharest, Romania; florentina.gatea@incdsb.ro; 3 Department of Genetics, ICUB-Research Institute of the University of Bucharest, 36-46 Bd. M. Kogalniceanu, 5th District, 050107 Bucharest, Romania; ionela.sarbu@bio.unibuc.ro (I.S.); diana.pelinescu@bio.unibuc.ro (D.P.)

**Keywords:** anti-inflammatory, butyric acid, curcumin, modulation, in vivo

## Abstract

The multiple causes of cardiovascular diseases signify a major incidence and developmental risk of this pathology. One of the processes accountable for this pathologic development is the instauration of dysbiosis and its connection with an inflammatory process. Low antioxidant colonic protection encourages the progression of inflammation, with cardiovascular dysfunctions being a secondary consequence of the dysbiosis. Curcumin is one of the bioactive compounds displaying promising results for the reduction of an inflammatory process. The present study aims at demonstrating the capacity of three extracts drawn from *Curcuma* (*C.*) *longa* through an in vitro simulation process, for microbiota modulation in patients with hypertension. The acidic pH in the extraction process determined a high curcumin content in the extracts. The major phenolic compound identified was curcumin III, 622 ± 6.88 µg/mL for the ethanol/water/acetic acid extract. Low EC50 values were associated (0.2 µg/mL for DPPH scavenging activity) with the presence of curcumin isomers. A metabolic pattern became evident because the relationship between the short-chain fatty acids acted as a clinical biomarker. The curcumin present stimulated the formation of butyric and propionic acids. Microbiota activity control included a high degree of curcumin degradation and biotransformation in the other phenolic compounds. This developmental process was supported by the progression in the enterobacteria with a corresponding escalation in the pH level. The metabolomic pattern demonstrated a performance similar to the administration of dietary fibre, with the positive effects being dose-dependent.

## 1. Introduction

Cardiovascular disease management is long-lasting, and involves several medications because in most cases it is allied to other pathologies [1]. However, in its treatment, the vegetable supplements (herbs, mushrooms, spices, etc.) act as a non-toxic alternative [2]. The development of chronic disease often starts with the occurrence of an inflammatory process that indirectly results in physiological dysfunctions [3]. Oxidative stress, among all the processes, is the chief culprit that determines the development of cardiovascular pathologies. In this context, the role of cerebral oxidative stress in the manifestation of hypertension has been demonstrated [4]. The interest shown towards and reasons for the selection of *Curcuma longa* in this study arises from certain health benefits, which result from its anti-inflammatory and antioxidant properties [5]. Besides, the curcumin isomers possess many biological activities, not clearly studied as yet, involving the interactions with human microbiota [6].

Further, the influence exerted by the microbiota on host homeostasis is the critical point of the brain-gut axis [7]. Thus, the interest in the complex action of the microbiota has escalated, with particular emphasis on the explicit characterisation of the fingerprint of the microbiota of patients with cardiovascular diseases. Further correlation of this aspect was done with the metabolomic profile. Specific biomarker molecules have been identified that are directly involved in the development of heart disease [8]. Due to the significant progress made in molecular biology, it is now possible to recognise the preventive risk factors, such as colon microbiota, that play a role in the development of cardiovascular diseases.

Interaction of microbiota with bioactive compounds (especially phenolics) from medicinal plants extracts is due to the large quantity that reaches the colon, but only a small part is absorbed in ascending segments of the human digestive tract [9]. There are few in vivo studies that directly show the effect of the microbiota pattern on the bioavailability of the polyphenolic fraction. By in vitro/in vivo correlation, it has been demonstrated that the biological effect is a selective one, characteristic of each subclass of phenolic compounds. This is the result of a series of biotransformations, which changes the expected effect [10]. Curcumin is an example in this regard because it is a well-tolerated compound, although there is little evidence in vivo. Meta-analyses of clinical uses have shown that administration has a positive effect in patients with irritable bowel syndrome, and microbiota modulation has been based on correlation with antioxidant and anti-inflammatory activities [11,12,13].

Thus, the dysbiosis begins with an inflammatory process promoted by the low antioxidant potential at the microbiota level [14]. Dysbiosis may be caused by any of the following, viz., genetic heritage, food consumption or the occurrence and progress of cardiovascular diseases [15]. However, the favorable strains, at the level of Bacteroides sp., govern the strong response during oxidative stress [16]. The study is based on the following findings: (i) Cardiovascular dysfunctions (in those patients with hypertension and dyslipidemia) are correlated and frequently determined by the microbiota dysbiosis, (ii) the decrease in the favorable strains may be connected to the development of cardiovascular risk, (iii) the metabolomic profile is vital to cardiovascular management, and (iv) the microbiota profile signifies a biomarker that can determine early intervention and thus reduce the risk of mortality [17]. The present study aimed at controlling the in vitro effect of the consumption of *C. longa* extracts on the microbiota of a target group, which included hypertensive patients. The research presents a metabolomic profile of the microbiota fingerprint modification, correlated with the in vitro and in vivo characterization of the extracts.

## 2. Materials and Methods 

### 2.1. Chemicals

Ethanol, methanol, acetic acid, glycerol, 2,2-diphenyl-1-picrylhydrazyl, Folin–Ciocalteu reagent, ascorbic acid, ferrozine disodium salt, ferrous chloride, ethylenediaminetetraacetic acid (EDTA), glucose, peptone, yeast extract, sodium chloride, hydrogen peroxide, sodium hydroxide, sodium tetraborate, methylene chloride, ferulic acid, vanillin, sodium dodecyl sulphate, hydrochloric acid, phosphoric acid, calcium carbonate, agarose, TBE buffer, and cetrimonium bromide were purchased from Sigma-Aldrich GmbH (Sternheim, Germany). Maltodextrin was purchased from Agnex, Bialystok, Poland (17% dextrose index). Peptone water and MRS broth media were purchased from Oxoid Ltd. (Hampshire, U.K.). All reagents were of analytical grade.

### 2.2. Extraction Process

Dried *C. longa* powder (Kotanyi Condimente SRL, Bucharest, Romania), was used to obtain the three extracts. The extraction was performed in Duran bottles (24 h, at room temperature under stirring) at a concentration of 1% turmeric with the following three solvent mixtures (*v*/*v*): (a) Ethanol/water = 50/50, (b) ethanol/water/acetic acid = 50/49.5/0.5, and (c) ethanol/acetic acid = 99.5/0.5 [18]. Subsequently, after the extraction process, the mixture was filtered under vacuum using Whatman filter paper no. 1 [19]. The extract was then mixed with 7% maltodextrin until total homogenisation was complete and freeze-drying achieved [17]. Moreover, before conducting the experiments, the dried extracts were dissolved, in 50% (*v*/*v*) ethanol, in a series of dilutions ranging from 0.01 to 0.1%. The freeze-drying process was performed in aseptic condition in order to eliminate the possible interactions with the in vitro research.

### 2.3. In Vitro Simulation Process

All the in vitro tests were performed in a single-stage culture system GIS1 simulator (http://gissystems.ro/gis-technology/). The in vitro colonic simulation system involved the use of three Duran borosilicate glass bottles (500 mL capacity) having a removable screw cap [20]. The principle of the GIS1 system operations has been described in a previous study [21], and was, utilised as a continuous fermentation process [20].

First, the hypertensive patients’ microbiome was reconstituted after 7.0 days mean interval and this process followed the protocol described earlier [22]. Volunteers between 40 years and 70 years of age were drawn from both sexes. Care was taken to ensure that these individuals had not received any treatment with antibiotics or other interfering drugs over the past 6.0 months, as these agents might alter the microbiome fingerprint. The samples (feces) were handled in accordance with the UASVM Bucharest ethical guidelines (ColHumB Registration number: 1418/23.11.2017; www.colhumb.com) and individually analyzed. Feces samples were collected twice from each volunteer and treated separately without any pooling (*N* = 3). Informed consent was obtained from each participant for the sample, storage after the collection. Samples were collected in 20% glycerol and stored at −15 °C until use [18,19]. Following the removal of large particles, the microbiota was reconstituted in peptone water [23]. Microbiota obtained (in the same conditions) from healthy persons was used as a control. The second control was obtained from the same hypertensive patients’ untreated with *C. longa* extracts (hpu control). 

First, the sampling, at the level of each colonic segment of the simulation process was accomplished, in the case of each product addition. Further, to examine the effect of the atomised extracts on the evolution of microbial communities, three identical series of treatments were performed by adding one dose (capsule) every 6.0 h in the in vitro simulator. Moreover, for the effective delivery of the products in the colon, enteric-coated capsules were used (size 0; BSC, Wenzhou, China) instead of direct addition of the extract. The capsules were directly added in the simulated environment under sterile conditions [17]. When the simulation was completed, each sample was centrifuged, (4000× *g*, 15 min, Hettich Universal 320, Hettich GmbH & Co., Kirchlengern, Germany) and the sediment (microbial fingerprint) was placed in glycerol 20% for qPCR analysis [20]. Control tests were run with ethanol, and capsules with maltodextrin only in order to establish whether solvent traces or lack extracts affect microbiota response. 

### 2.4. Antioxidant Activity Quantification

#### 2.4.1. In Vitro Analysis

To determine the antiradical potential, the DPPH (2,2-diphenyl-1-picrylhydrazyl) radicals were utilised according to the spectrophotometric protocol, for which the readings of the optical density of the reaction mixtures at 517 nm Helios γ (Thermo Fisher Scientific, Waltham, MA, USA) were also required. The antiradical potential was expressed in terms of the effective concentration (EC50), which was the extract concentration (μg/mL) required to inhibit the DPPH activity by 50% after at least 30 min of incubation [24]. Ascorbic acid was used as positive control. 

Subsequently, the reduction power was determined spectrophotometrically at 700 nm wavelength (Helios γ, Thermo Fisher Scientific, Waltham, MA, USA), with the results being expressed as the value of the extract concentration (μg/mL) necessary to obtain an optical density of 0.5, at the specified wavelength [25]. Ascorbic acid was used as a positive control.

Further, the chelating activity was determined by using ferrozine (5 mmol/L) as the reagent. The absorbance was spectrophotometrically measured at 562 nm (Helios γ, Thermo Fisher Scientific, Waltham, MA, USA). The chelating activity was expressed as the effective concentration (EC50), which represented the concentration of the extract (μg/mL) required to obtain a 50% value [26]. EDTA was used as a positive control.

The choice of methods to evaluate the antioxidant activity was performed according to the reaction mechanism and the existing reference data [27,28,29].

#### 2.4.2. In Vivo Antioxidant Potential

To determine the antioxidant potential in vivo, a modified protocol of dos Santos Andrade et al., 2011 [30] was employed. *Saccharomyces* (*S.*) *boulardii*, a probiotic yeast obtained from the University of Lille, Lille, France, was used as the in vivo model. The biomass was obtained using the YPG medium (2% glucose, 2% peptone, and 1% yeast extract) and further cultivated in the lab shaker incubator at 30 ° C, for 48 hh, at 150 rpm. The yeast cells were separated through centrifugation at 4500× *g* for 5 min and washed twice with NaCl 0.9% (sterile). Finally, the yeast cells were diluted with the same solution, whose optical density (OD) was 1.0 at 600 nm [31]. Further, to obtain the critical concentration, which is defined as the cross between the viability and mortality lines, the following mixture was used: 0.1 mL sample, 0.1 mL yeast cells in a sterile saline solution, and 0.2 mL H_2_O_2_ (different concentrations–0.001%, 0.005%, 0.01%, 0.2%, 0.3%). The mixture was made on a honeycomb plate and incubated at 30 °C for 1 h by using Bioscreen C MBR (Oy Growth Curves Ab Ltd., Helsinki, Finland). The cells were diluted by serial dilutions, then plated on a solid YPG and finally incubated for 48–72 h at 30 °C in an incubator (Memmert Model 100-800, Memmert GmbH & Co., Schwabach, Germany). The results were expressed as a percentage of the viability and mortality, through the use of a control sample without extract as a protection against oxidative stress [32].

### 2.5. Metabolomic Profile.

#### 2.5.1. Quantification of the Curcuminoids

The experiments were performed with an Agilent capillary electrophoresis (CE) instrument equipped with a diode array detector (DAD) and CE standard bare fused silica capillary (Agilent Technologies, Germany) with a 50 μm internal diameter and 72 cm effective length. Prior to use, the capillary was washed successively with basic solutions: 10 min with 1 N NaOH, 10 min with 0.1 N NaOH, followed by ultrapure water for 10 min, and running buffer for 20 min. The capillary was flushed between runs with 0.1 M NaOH for 1 min, H_2_O for 1 min, and background electrolyte (BGE) for 2 min. Moreover, after three consecutive runs, the BGE was refreshed. Data acquisition and processing were performed with ChemStation software. Sample injection was performed using the hydrodynamic mode (35 mbar/12 s), while the capillary was maintained at a constant temperature of 300 C.

The method selected to quantify the curcuminoids is based on Yuan and Weng (2005) with some variations [33]. The separation of the curcuminoid compounds was obtained using 15 mM tetraborate buffer, pH = 10.64 (adjusted with 1 M NaOH) as the background electrolyte. The BGE was filtered through 0.2 μm membranes (Millipore, Bedford, MA, USA) and degassed before use. Using 30 kV voltage and direct UV-Vis absorption, detection was performed from 200 nm to 450 nm, with the samples being quantified at 262 nm.

##### Sample Preparation 

First, 2 mL of each initial extract (ethanol/water; ethanol/water/acetic acid; ethanol/acetic acid) was vortexed with 15 mL of methylene chloride for 5 min and subsequently centrifuged for 10 min at 6000× *g* at 4 °C. The organic layer was separated and evaporated to dryness in a nitrogen atmosphere. The residue was dissolved in 250 µL methanol and analyzed by capillary electrophoresis. Similarly, 5 mL of each sample resulting from colon simulation was processed and the residue which was taken in 100 µL of methanol was subsequently analysed by CE.

#### 2.5.2. Ferulic Acid and Vanillin Quantification by CE

The ferulic acid and vanillin in the samples obtained after colon simulation were analyzed using a previously published method [34]. The analysis was done with an Agilent CE instrument equipped with a diode array detector (DAD) and CE standard bare fused silica capillary (Agilent Technologies, Waldbronn, Germany) having an internal diameter of 50 μm and an effective length of 72 cm. As a migration electrolyte (BGE), 45 mM tetraborate buffer was used with 0.9 mM sodium dodecyl sulphate (SDS), adjusted to a pH of 9.35 with 1 M HCl. The method required the use of 30 kV voltage, constant temperature of 300 °C, and direct UV absorption at 280 nm.

Sample preparation: First, 15 mL of each sample was purified through solid phase extraction with a C18 cartridge (Bond Elut Plexa, Agilent, Waldbronn, Germany). The cartridge was preconditioned with methanol (10 mL) and washed with water (5 mL), after which the sample was applied. After the sample passed through, the cartridge was washed with 5 mL water and finally with 5 mL methanol (solvent for the extraction of the polyphenols). The methanolic effluent thus collected was concentrated to 1 mL, filtered through 0.2 μm membranes (Millipore, Bedford, MA, USA) and degassed before injection. Standard addition was further employed, to evaluate the extraction efficiency.

#### 2.5.3. Organic Acids Analysis by CE

The organic acid analysis was done based on the earlier published method [35]. A standard bare fused silica capillary (Agilent Technologies, Waldbronn, Germany) with an internal diameter of 50 μm and an effective length of 72 cm was used. The migration electrolyte (BGE) was 0.5 M H_3_PO_4_, 0.5 mM of CTAB (pH adjusted with NaOH at 6.24), and 15% vol. of methanol as an organic modifier. In this method 25 kV voltage was applied at a constant temperature of 25 °C, and direct UV absorption at 200 nm. The samples obtained after colon simulation were filtered through 0.2 μm membranes (Millipore, Bedford, MA, USA) and degassed prior to injection.

### 2.6. The qPCR Profile of the Microbiota

After the samples passed through the GIS1 colon simulator, quantification of the microbial community was done. Further, the principal bacterial groups from the human gut were analyzed using the quantitative polymerase chain reaction (qPCR) technique. Specific primers for phylum Firmicutes, the Bacteroides-Prevotella-Porphyromonas group, Enterobacteriaceae family, Lactobacillus-Lactococcus-Pediococcus group, and genus Bifidobacterium have been already revealed in earlier studies. A bacterial universal primer pair was used to determine the bacterial load from each sample [21].

The DNA was extracted from 1 mL of the sample using the PureLink™ Microbiome DNA Purification Kit (Invitrogen, Waltham, MA, USA), while the DNA concentration and purity were measured with the NanoVue Plus spectrophotometer (GE, Boston, MA, USA).

The qPCR analysis was conducted on a 7900 real-time PCR equipment (Applied Biosystems, Foster City, CA, USA) using the Power SYBR Green PCR Master Mix (Applied Biosystems, Waltham, MA, USA) and 40 ng of the DNA template was introduced in each reaction. Based on the results obtained after the amplification reaction optimisation, the primer concentration was between 0.2–0.5 µM [20]. 

The reference strains: *Escherichia* (*E.) coli* ATCC 10536, *Lactobacillus (L.) plantarum* ATCC 8014, *Bifidobacterium (B.) breve* ATCC 15700, *B. fragilis* DSM 2151, and *Enterococcus (E.) faecalis* ATCC 51299 were used for the standard curve. All the samples were run in triplicate [21].

### 2.7. Phylogenetic Diversity of the LAB Strains

Dilutions from each sample were cultured on the MRS + CaCO_3_ plates for 48 h at 37 °C. Five colonies were randomly selected from the plate and cultivated in 1 mL MRS broth for 24 h at 37 °C. The cultures were centrifuged for 5 min at 10,000× *g*, followed by a one-time wash with sterile distilled water (SDW) with subsequent resuspension in 400 µL SDW, and frozen for 1 h at −70 °C. The PCR Master Mix (Promega, Madison, WI, USA), 0.2 µM of the primers (5’-CTG CTG CGT CTG CTG-3’), and 1 µL of the lysate culture were introduced in a 25 µL PCR reaction. The PCR amplifications were done in Mastercycler Nexus (Eppendorf, Hamburg, Germany). The amplification programme involved initial DNA denaturation for 7 min at 95 °C, 30 cycles of DNA denaturation for 1 min at 94 °C, annealing for 1 min at 53 °C, an extension for 8 min at 65 °C, and a final incubation for 16 min at 65 °C. 

The PCR products were separated in 1.7% agarose gel electrophoresis with 1X TBE buffer. Migration was performed at 65 V for 2 h.

The genetic profiles were analyzed with PyElph 1.4 using the WPGMA clustering method. *L. plantarum* ATCC 8014, *E. faecalis* ATCC 51299, *Lc. lactis subsp. lactis* DSM 20729, and *L. acidophilus* ATCC 314 were used as the reference strains [21].

### 2.8. Statistical Analysis

Evaluations of all the parameters investigated were performed in triplicate, with the results expressed as the mean ± standard deviation (SD) values of three observations. The mean and SD values were calculated using the IBM SPSS Statistics 23 software package (IBM Corporation, Armonk, NY, USA). To do the calculations, the significance level was set at: Significant = *p* ≤ 0.05; very significant = *p* ≤ 0.01; and highly significant = *p* ≤ 0.001 using the normal distribution of the variables. The differences were analyzed by ANOVA followed by a Tukey post hoc analysis. The IBM SPSS Statistics software package (IBM Corporation, Armonk, NY, USA) was used to analyze and correlate the experimental data [18].

### 2.9. Sample Availability

Samples of the three extracts are available from the corresponding author (in certain conditions).

## 3. Results

### 3.1. The In Vitro and In Vivo Antioxidant Activity

**Figure 1:**
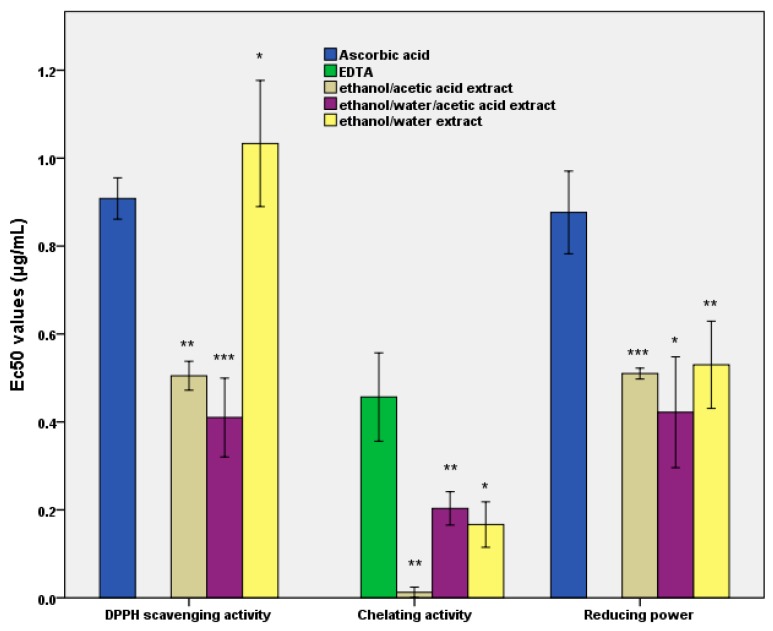
The figure represents the effective concentration (EC50) values for in vitro antioxidant activity as a measure of the impact of curcuma extract after the simulations in the GIS1 system. Different letters mean significant statistical differences ((* = *p* ≤ 0.05; ** = *p* ≤ 0.01; *** = *p* ≤ 0.001), *n* = 3).

Figure 1 depicts the in vitro antioxidant activity of all the three extracts. These determinations revealed that the capacity of the extracts to respond via biological activities is directly dependent upon the presence of the principal compound. The results also demonstrated the lowest EC50 value for the ethanol/water/acetic acid extract. Further, from the Figure it is evident that the antiradical activity was the lowest with an EC50 of a maximum of 1 μg/mL when ethanol/water was used as the solvent. By contrast, this value was around 50% higher than when an acidic extracting medium was used (Figure 1). 

The EC50 values of chelating capacity were lower by more than 50% compared to the control, behavior recorded for all three extracts. Ethanol/water/acetic acid extract had an EC50 value of 0.20 μg/mL, *p* < 0.01. The results demonstrated that the lack of water did not determine the presence of compounds that can increase chelating capacity in vitro. For the reduction power, the values were relatively equal, with a minimum of 0.4 μg/mL (*p* < 0.05) for the ethanol/water/acetic acid extract. This value, 20% lower than the rest of the extracts, was obtained in the absence of water as a solvent.

**Figure 2:**
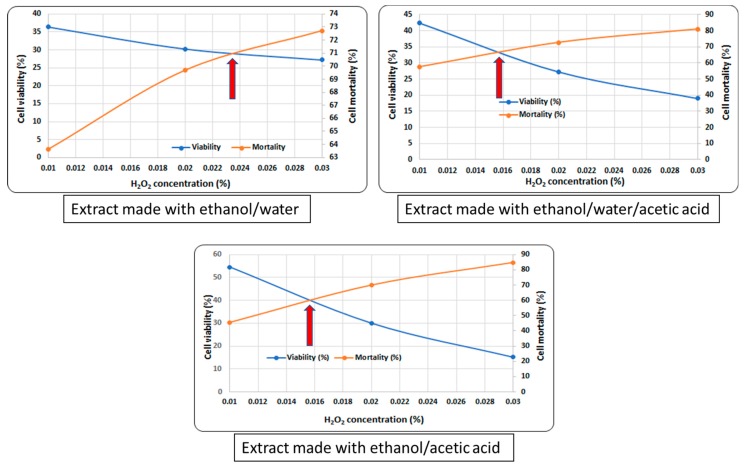
The cell viability as a parameter of the in vivo antioxidant activity in the presence of *C. longa* extracts.

Figure 2 represents the in vivo activity. The critical point (red arrow in Figure 2) was marked as indicated by the use of the graphics model. The acid extraction, however, determined a similar level of peroxide concentration at which the strain tested was able to survive. In fact, it was observed to be approximately 30% lower than the first solvent and is explicably based on the concentration of the extracted components. Thus, the acetic acid determined the presence of compounds which solubilize only in this medium and which have raised the degree of resistance to oxidative stress.

### 3.2. Determination of the Quantities of Curcumin, Ferulic acid, and Vanillin 

**Figure 3:**
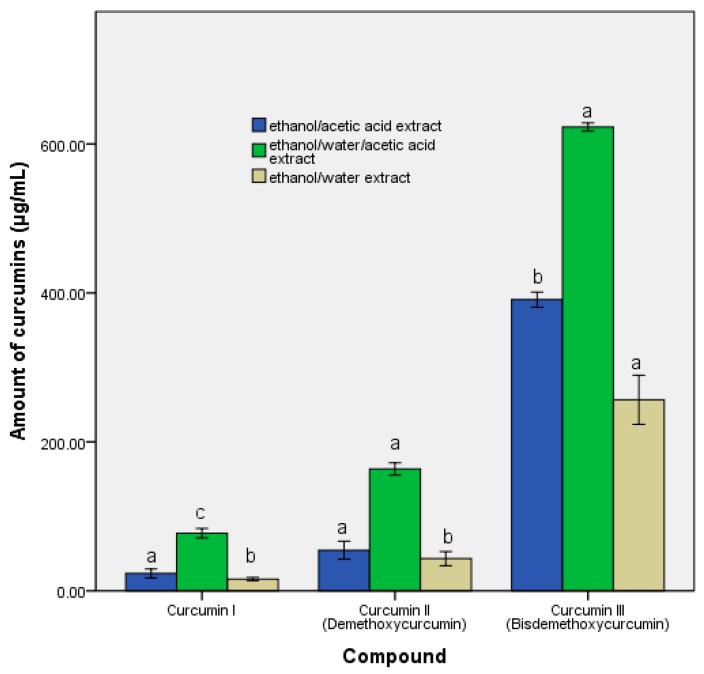
The total amount of curcumins in the three extracts. Within each group of samples, different letters mean significant statistical differences ((a = *p* ≤ 0.05; b = *p* ≤ 0.01; c = *p* ≤ 0.001), *n* = 3).

Predominantly, curcumin III was found to be the major constituent in the acetic acid-based extractions. For the three-solvent extract, the level was 622.5 μg/mL, around 35% more than the presence of only ethanol and acid. Although curcumin is water insoluble, the presence of water determined the solubilisation of the other compounds, which indirectly assisted in the release of the curcumin isomers from the substrate. Besides, the extracts also contained other compounds, although they were in quantities that did not directly affect the in vitro study (Figure 3). According to the earlier data, the hydroalcoholic extract determined the presence of the significant oleoresins, which explained the EC50 values [36].

Curcumin degradation products (ferulic acid or/and vanillin) [37] were not identified in any of the samples, which in turn led to the assumption that curcumin and its derivatives had been metabolised by the microbiota.

### 3.3. Microbiota Fingerprint Response

Some food ingredients directly impact human health by their capacity to modify the gut composition. In the present study, we analysed the impact of the curcuma extract on the main microorganism groups from the human gut. According to the spectrophotometric analysis, all the DNA extracts had a concentration of over 150 ng/µL and were uncontaminated by proteins or RNA, thus being suitable for qPCR (data not shown).

**Figure 4:**
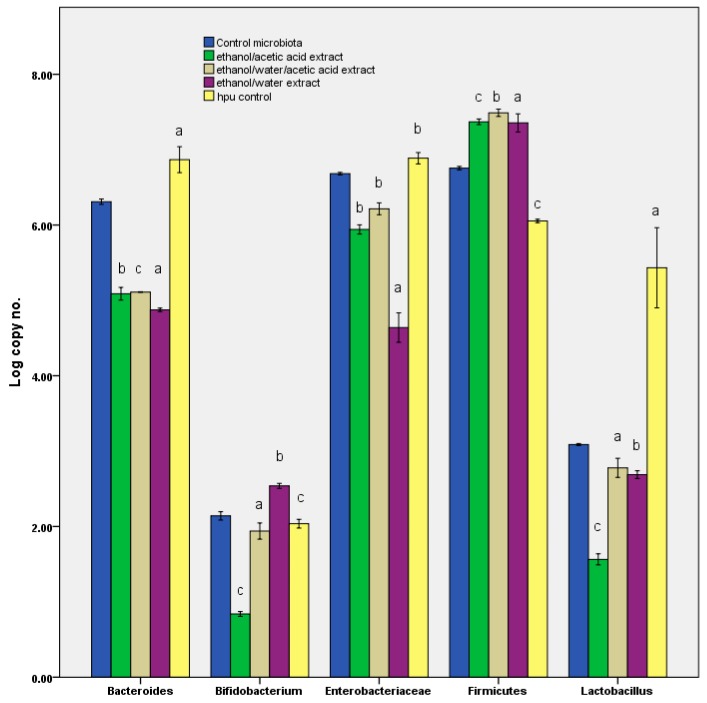
Log of number of copies obtained after in vitro tests through GIS1 as a measure of the impact of curcuma extracts on the number of the main groups of microorganisms from human gut. Different letters mean significant statistical differences ((control microbiota vs. treated samples/hpu; a = *p* ≤ 0.05; b = *p* ≤ 0.01; c = *p* ≤ 0.001), *n* = 3).

As shown in Figure 4, the curcumin extract strongly influences the Enterobacteriaceae group although it affects the Bacteroides-Prevotella-Porphyromonas group to a lesser degree, where the number of the cells remains constant in all the samples. In the ethanol/water extract, the number of Enterobacteriaceace is 10 times lower than it is in the remaining samples.

At gram-positive bacteria for phylum Firmicutes, which is also the dominant group from the microbiota, the number of cells is higher than control (10^7^ genomes/mL), but it is constant among the samples, instead the dynamics within this phylum is different. Therefore, the number of microorganisms in the Lactobacillus-Lactococcus-Pediococcus group is approximately 10 times higher in the ethanol/water extract and ethanol/water/acetic acid extract samples than in the ethanol/acetic acid extract. The number of bifidobacteria is also higher in the first two samples analyzed in the ethanol/water extract and ethanol/water/acetic acid extract.

#### 3.3.1. Phylogenetic Diversity of the LAB Strains

**Figure 5:**
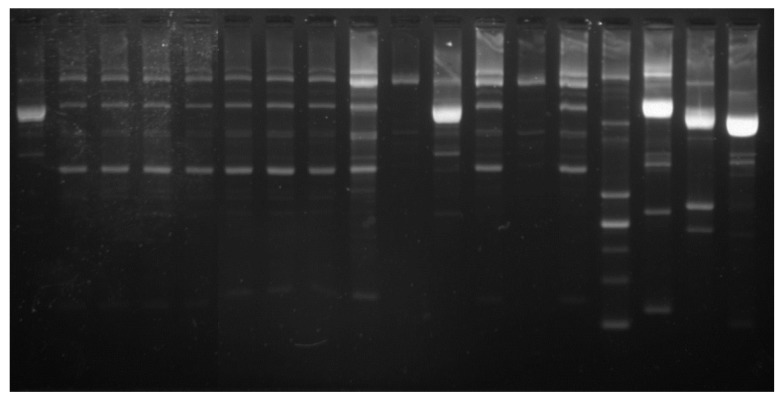
The rep-PCR profile of lactic acid bacteria (LAB) strains obtained after in vitro tests through GIS1 system as a measure of the impact of curcuma extracts. 1–14 new LAB strains, 15- L. acidophilus ATCC 314; 16- L. plantarum ATCC 8014, 17- Lc. lactis subsp. lactis DSM 20729, and 18- E. faecalis ATCC 51299 (from left to right).

LAB (lactic acid bacteria) strains represent an important microbe group from the human gut, which, several times, has been found in association with host health. As this group includes both beneficial and pathogenic strains, we have analyzed the phylogenetic relations of the strains from each sample by rep-PCR (Figure 5).

Different colonies from the MRS plates were selected and analyzed based on their phylogenetic profile using the PyElph 1.4 program. The phylogenetic analysis revealed the presence of one dominant clone in the ethanol/water extract and ethanol/water/acetic acid extract, which is closer to the phylogenetic on *L. acidophilus* ATCC 314. In the ethanol/acetic acid extract, two different clones were identified, which are part of the same cluster, with one being identified in the other two samples.

#### 3.3.2. Metabolomics: Response of the Microbiota

After the curcuma extracts were administered, the metabolic activity revealed variations between the samples according to the concentrations of the organic acid content belonging to the same microbiota. The variations could be explained by the difference between the microbiota fingerprints and by the curcumin level. Further, the relation between the propionic and butyric acid represented a microbiota modulation biomarker (see Table 1). Curcuma extracts, by the presence of curcumin (see Figure 3), stimulated the formation of butyric and propionic acid in the ethanol/water/acetic acid extract and ethanol/acetic acid extract. This behaviour was similar to the administration of dietary fibre. 

Also, lack of propionic acid was registered when ethanol/water extract was administered. This diminished pattern of short-chain fatty acids (SCFAs) was also characterized by the smallest amounts of butyric and acetic acids, compared with control microbiota. The high level of lactic acid was correlated with the metabolic activity that increased in the presence of curcuma extracts. In addition, in vitro SCFAs production by the two inocula (Table 1) demonstrated a low microbiota metabolic response compared to treated microbiota.

## 4. Discussion

The study tested the hypothesis that hypertensive patients have dysbiosis at the microbiota level, based on the progression of an inflammatory process associated with oxidative stress activity. Results have proved that the *C. longa* extracts were associated with a reduction in the oxidative stress effects, modification of the microbiota pattern, and improvement in the level of biomarkers (like butyric acid formation). Further, the in vitro antioxidant effects were not directly correlated with in vivo activity, although the data were relevant for the observation that low EC50 values were linked to the presence of the curcumin isomers. 

**Figure 6:**
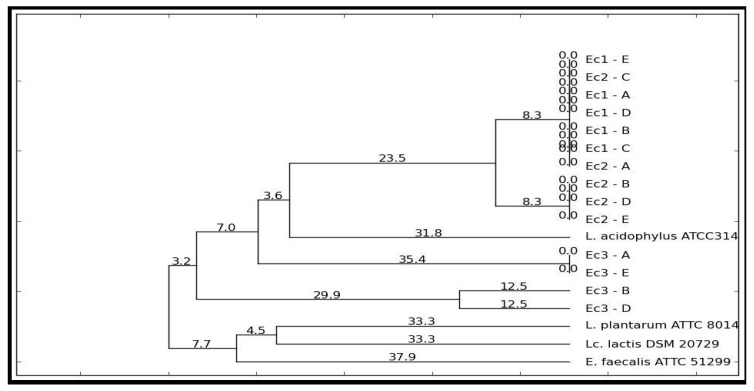
Dendrogram of LAB strains using WPGMA clustering method obtained after in vitro tests through GIS1 system as a measure of the impact of curcuma extracts. EC1 = ethanol/water extract; Ec2 = ethanol/water/acetic acid extract; Ec3 = ethanol/acetic acid extract.

On comparing the response of the *S. boulardii* cells, the role of specific compounds (especially curcumin) in combating oxidative stress became clear. The critical point indicated the potential of the ethanol/water/acetic acid extract to support the physiological mechanisms of the inhibition of the free radicals [32]. Such analysis may be an easy method of evaluating the effectiveness of a nutraceutical in modulating the response of the human microbiota and intervening in the improvement of inflammatory processes. The coevolution of the microbial pattern with the host’s health was supported by the data displayed in Figure 6. The phylogenetic relationships after the administration of the extract were an indicator of the role of the bioactive compounds in the expression of the metabolomic biomarkers (see Table 1). Stimulating bacterial diversity was a factor that promoted the reaction to the inflammatory process. Thus, it can be deduced that the action of oxidative stress induced differences in the microbial pattern [20] through a direct link to the presence of the major bioactive compounds (see Figure 3). Thus, such an extraction pattern expressed a distinct in vitro/in vivo behaviour based on the difference in solvent and the bioavailability of the principal bioactive compound (curcumin). The in vivo response, compared to the in vitro, revealed a pattern that is in direct correlation with the pH of the solvent used.

The in vivo response of the *S. boulardii* eukaryotic cell represents the preclinical assessment of the enzymatic mechanisms of hydrogen peroxide reduction as an indicator of the development of degenerative pathologies [38]. The reduction in the accumulation of the oxidised proteins as an effect of the stress caused by hydrogen peroxide decomposition confirms the protection given by the curcumin against the development of degenerative pathologies [39]. These data were synchronised with a low EC50 value for the inhibition of lipid peroxidation (see Figure 1). They also revealed a decrease in oxidative damage, confirmed by the value of the critical point in vivo (see Figure 2), particularly for the use of ethanol/water/acetic acid as the solvent. 

However, post in vitro simulation, the curcumins identified were absent. This finding could be explained in several ways because trace amounts were found in the other two compounds in which it became transformed. The first cause concerns biotransformation determined by the fermentative activity of the microbiota [11]. It has been noted as well that the low alkaline pH is indicative of high solubilisation and a high bioconversion rate in the other compounds [11] or utilisation as a carbon source [40]. In this context, the earlier research revealed that the curcumin could act as a carbon source for Enterobacteriaceae. In parallel, besides the extracts, a sample containing ethanol alone was run; it was evident that it had exerted no effect on the microbiota pattern. The same effect was observed after the blank sample (capsule + maltodextrin without curcuma extract; Figure 4). This behaviour can thus be interpreted as a response to the curcumin metabolism, which translates into the action of the compounds produced through its degradation. Thus, the pH value, in response to the curcumin action, can be used as a biomarker, which is recognised as a parameter by both the present investigation and prior studies [41]. These results confirmed a previous study (https://aor.ca/blog/curcumin-under-fire-the-root-of-the-problem), which proves the instability of this compound. Based on the highly degrading behaviour it was possible to explain the contradictions observed in this study. Besides, the results could partially explain the pharmacological target of curcumin based on the interaction with the microbiota fingerprint through the metabolomic pattern [6].

Thus, the administration of the extracts was similar to a polypharmacological effect. The in vitro administration was mediated by the degradation products of curcumin, demonstrating a multifactorial action [42]. The fermentative action of the microbiota showed that its low stability induced a multiple, microbiological, and metabolomic response, which was in accordance with the previous studies [43]. Reduced quantities of vanillin and ferulic acid have not been identified as significant degradation compounds. They could also represent biomarkers of the pharmacological action of the *C. longa* extracts [44]. The results of this study highlighted the bioavailability of the curcumin [43]. Pharmacological action was performed by the metabolomic indicators and not directly through increased bioavailability. Thus, this study confirms the impact of the modulating action of the curcumin on the perturbed microbiota. The metabolomic pattern and increase in the microbial diversity (LAB strains) were a pharmacodynamic response of the *C. longa* extracts, which was a good indicator of the physiological and biochemical effects.

The aim of the article was achieved as it demonstrated a correlation between the metabolic products and microbial pattern modulation in the simulated colon (see Table 1). The increase in the quantity of curcumin III from one extract to another was not correlated with the acidity of the solvent (ethanol/acetic acid) [45,46], but can be explained by the reduced acetate/propionate ratio. Moreover, it was found to be around 20%, which corresponds to a progressive decrease in the serum cholesterol [26]. The reduction in cardiovascular risk is a confirmation of the high rate of biotransformation of the phenolic component and favourable microbial proliferation. Thus, the modulation of the microbial pattern can correct the progression of the inflammatory processes of the host, thus reducing the sensitivity to oxidative stress. The reduction in the oxidative stress disorder naturally improves the cardiovascular risk, in particular, maintaining hypertension within optimal limits [30].

The modulation of the metabolomic pattern, by improving the ratio of the SCFAs, showed that the ethanol/water/acetic acid extract has potential therapeutic use against cardiovascular progression. By raising the quantity of curcumin, prebiotic activities were induced, by a modulation of the intestinal microbiota and specific metabolic pathways, contributing towards host health improvement. This effect is similar to that of green tea polyphenols [31]. Clinical data also showed that an increase in the butyrate level, caused by the use of functional products, exerted a positive effect. The presence of curcumin in the in vitro environment (see Figure 3) was correlated to the rise in the butyrate content, particularly in the acid extractions (see Table 1). The level of butyrate plays an important role in responding to different inflammatory processes. Changing the SCFAs profile by stimulating the synthesis of butyrate correlated with curcumin administration [47]. This aspect represented a novelty of the study, demonstrating the altered microbiota response to the presence of curcumin. Even if the direct influence on the microbial pattern was balanced among the three extracts, the quantity of curcumin and the quantitative distribution between the isomers influenced the outcomes of the study. Thus, the differences between the isomers have also been translated by a modulation of the metabolic response of the microbiota. This is a significant factor in preclinical studies because the presence of various forms of the biomarkers plays a crucial role in reconstituting the pattern of the microbiota in hypertensive patients [33].

Also, an increase in the number of organic acids in the simulated environment was a health status indicator in accordance with a previous study [37]. This behaviour was correlated with the curcumin level and represented an indicator of an anti-inflammatory response [48].

## 5. Conclusions

Our study proved that the inhibitory concentrations towards more strains were reached by the administration of curcuma extracts. The in vitro experiments proved that the poor bioavailability does not mean that the curcumin was not metabolized by the gut bacteria. The effects of the *C. longa* extracts were correlated with the antioxidant potential and fingerprint microbiota modulation. The results showed a decrease in the microbiota dysbiosis for the ethanol/water/acetic acid extraction. The large curcumin quantity was correlated to a decrease in the unfavorable strain. The property of the curcuma extracts in reducing the oxidative stress is dependent upon their ability to enhance the antioxidant potential at the colon level. Valorisation of the favorable strains will result in a lowered inflammatory process, which is dose-dependent.

## Figures and Tables

**Table 1:** Organic acids levels (µg/mL) obtained after in vitro tests through the GIS1 system as a measure of the impact of curcuma extracts (administered as capsules) associated with the microbiota metabolic response.

Organic Acids (µg/mL)	Control Microbiota	Hpu Control	Treated Microbiota from Patients with Hypertension		
			Ethanol/Water Extract	Ethanol/Water/Acetic Acid Extract	Ethanol/Acetic Acid Extract
**Formic acid**	34.21 ± 5.35	nd	284.8 ± 21.07 ^a^	362.12 ± 15.00 ^a^	301.01 ± 9.00 ^b^
**Oxalic acid**	nd	nd	7.68 ± 0.43 ^b^	5.75 ± 0.40 ^a^	9.38 ± 0.51 ^a^
**Succinic acid**	nd	nd	62.04 ± 2.21 ^b^	39.54 ± 7.05 ^c^	31.4 ± 0.45 ^c^
**Malic acid**	nd	nd	nd	nd	20.48 ± 3.19
**Tartaric acid**	nd	nd	nd	nd	nd
**Acetic acid**	435.13 ± 6.23	340.50 ± 4.70 ^c^	505.16 ± 48.54 ^a^	918.97 ± 22.98 ^a^	1147.59 ± 73.88 ^a^
**Citric acid**	nd	nd	nd	nd	nd
**Propionic acid**	145.46 ± 3.45	180.50 ± 0.01 ^b^	nd	33.53 ± 3.77 ^b^	62.53 ± 3.69 ^c^
**Lactic acid**	330 ± 5.10	390.00 ± 5.60 ^c^	534.89 ± 32.60 ^c^	415.49 ± 11.32 ^a^	449.17 ± 12.53 ^d^
**Butyric acid**	146.65 ± 4.37	37.80 ± 0.76 ^c^	117.33 ± 4.68 ^b^	322.76 ± 11.59 ^a^	247.11 ± 29.84 ^a^
**Benzoic acid**	1.71 ± 0.10	nd	5.72 ± 0.56 ^c^	6.44 ± 0.30 ^b^	11.48 ± 1.06 ^a^
**Phenyllacticacid**	17.6 ± 0.36	0.55 ± 0.01 ^c^	3.48 ± 0.34 ^c^	4.27 ± 0.08 ^c^	2.84 ± 0.14 ^c^
**OH Phenyllactic acid**	44.58 ± 0.76	40.00 ± 1.80	2.28 ± 0.10 ^b^	10.88 ± 0.54 ^c^	2.25 ± 0.22 ^b^

^a^ = *p* ≤ 0.05; ^b^ = *p* ≤ 0.01; ^c^ = *p* ≤ 0.001, for control microbiota vs. treated samples/hpu, *n* = 3; nd—not detected.
